# Amyloid and the origin of life: self-replicating catalytic amyloids as prebiotic informational and protometabolic entities

**DOI:** 10.1007/s00018-018-2797-9

**Published:** 2018-03-17

**Authors:** Carl Peter J. Maury

**Affiliations:** Department of Medicine, University of Helsinki, Helsinki, Finland

**Keywords:** Origin-of-life theory, Amyloid world, RNA world, Prion, Molecular evolution, Primordial genetics

## Abstract

A crucial stage in the origin of life was the emergence of the first molecular entity that was able to replicate, transmit information, and evolve on the early Earth. The amyloid world hypothesis posits that in the pre-RNA era, information processing was based on catalytic amyloids. The self-assembly of short peptides into β-sheet amyloid conformers leads to extraordinary structural stability and novel multifunctionality that cannot be achieved by the corresponding nonaggregated peptides. The new functions include self-replication, catalytic activities, and information transfer. The environmentally sensitive template-assisted replication cycles generate a variety of amyloid polymorphs on which evolutive forces can act, and the fibrillar assemblies can serve as scaffolds for the amyloids themselves and for ribonucleotides proteins and lipids. The role of amyloid in the putative transition process from an amyloid world to an amyloid–RNA–protein world is not limited to scaffolding and protection: the interactions between amyloid, RNA, and protein are both complex and cooperative, and the amyloid assemblages can function as protometabolic entities catalyzing the formation of simple metabolite precursors. The emergence of a pristine amyloid-based in-put sensitive, chiroselective, and error correcting information-processing system, and the evolvement of mutualistic networks were, arguably, of essential importance in the dynamic processes that led to increased complexity, organization, compartmentalization, and, eventually, the origin of life.

## Introduction

It is generally believed that life on Earth passed through an RNA-world era in which RNA or an RNA-like polymer performed both informational and catalytic functions [1–4]. It is, however, unlikely that a functional ribonucleotide polymer could have existed under early Earth conditions [5–7]. The amyloid world hypothesis of the origin of life [8] posits that during that early period, about 4 billion years ago, peptide amyloids were the first molecular entities that were able to self-replicate, transmit information, and evolve. The model has gained momentum from recent empirical and theoretical studies clarifying the molecular mechanisms underlying amyloid formation, amyloid-related catalysis, and protein-encoded information processing. Here, I examine the model in light of recent advancements, develop the model further, and broaden the perspective to include an outline how a transition from a primitive β-sheet-based replicator world to a complex amyloid–ribonucleo-protein world might have occurred.

## The amyloid replicator

**Fig. 1:**
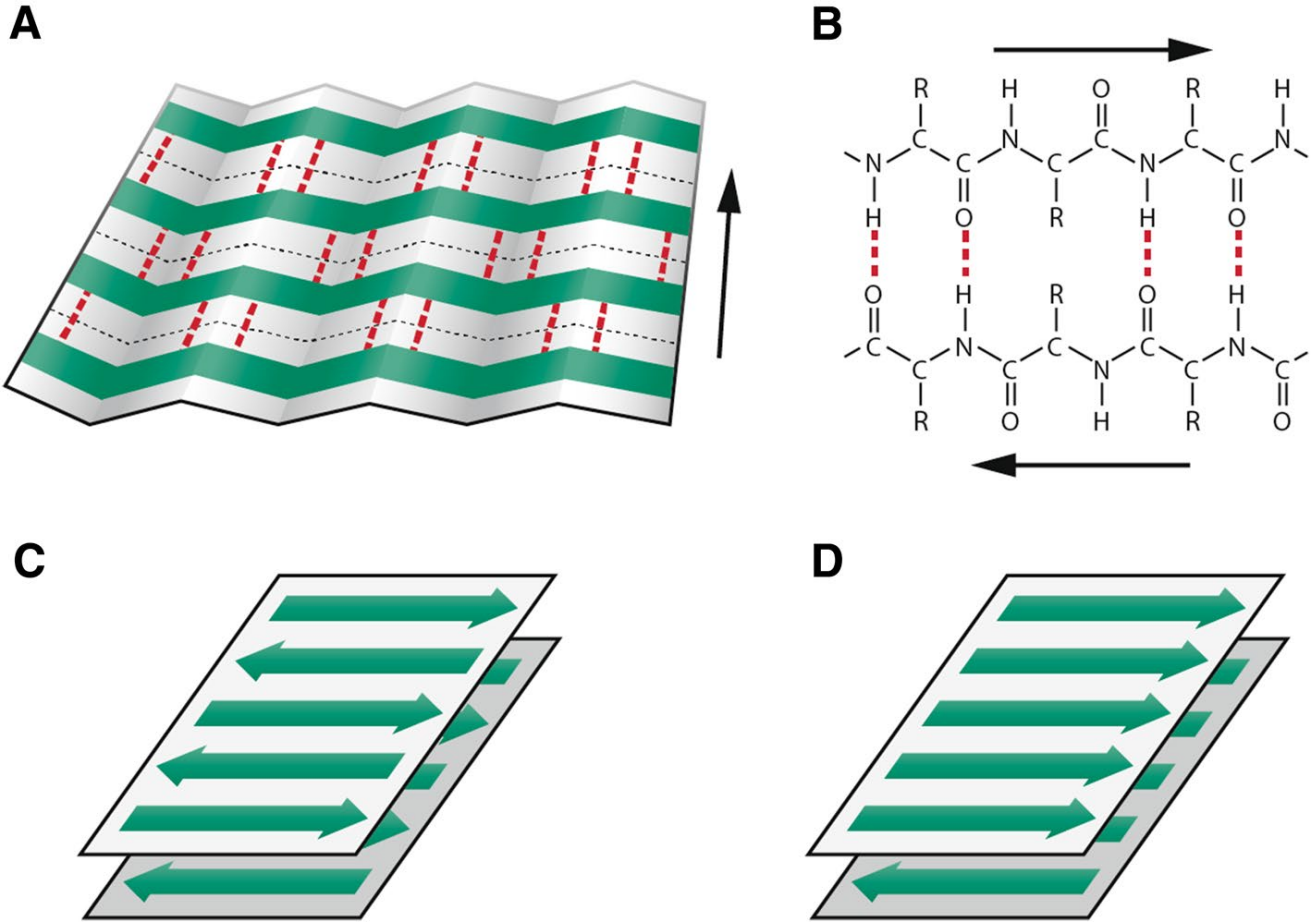
Schematic representation of the β-sheet structure of amyloid. **a** Section of a β-pleated sheet. The β-strands, which run perpendicular to the long axis of the fibril, are marked in green and interstrand hydrogen bonds in red. **b** Hydrogen-bonding pattern of two antiparallel β-strands. **c** Antiparallel bilayered β-sheet. **d** Parallel bilayered β-sheet. Typically, the repeating unit of the amyloid fibrils consists of two tightly packed layers of β-sheets with side chains within the bilayers forming a dry interdigitating zipper interface. The zippers differ in the organization of the β-strands within and between the β-sheets and in the stacking of the β-sheets enabling the formation of a diversity of structural variants. The cross β-structure gives rise to a characteristic X-ray diffraction pattern with a meridional reflection at 0.48 nm and an equatorial reflection at about 1.0 nm. These reflections correspond to the interstrand and intersheet spacings, respectively. The mature amyloid fibril is a highly ordered linear supramolecular structure forming long unbranched fibrils ranging from 5 to 12 nm in diameter. References are given in the text

Within the framework of origin-of-life research, the question of the mechanism of replication and information transfer is crucial. Given the stability and functionality problems connected with the RNA-world hypothesis [5–7, 9], the view that some other type of informational system probably preceded the RNA-based one has gained an increasing attention. According to the amyloid world hypothesis, the primordial information system was based on structurally stable catalytic and self-replicating β-sheet amyloid conformers [8, 10, 11]. The basis for the model is the conformational arrangement of the amyloid fold (Fig. 1). The cross-β sheet structure of amyloid, in addition to providing remarkable stability, can convey multifunctionality to peptides [12–26]. Even very short peptides may express diverse catalytic, replicative, and informational properties when adopting the amyloid conformation. This is in contrast to native peptides, which are easily denatured under harsh conditions, and whose functionality requires longer peptide sequences, the synthesis of which, again, would require an existing metabolic apparatus. Thus, under early Earth conditions, the amyloid fold would, obviously, have provided a substantial advantage for the survival and propagation of prebiotic peptides.

## Encrypting environmental information

Recent research has clarified many aspects of the molecular mechanisms underlying the amyloid-mediated information system. It has become evident that the coding element is the steric zipper structure of the amyloid motif and that recognition occurs by amino acid side chain complementarity [13, 18]. In the encryption process, environmental information is encoded in the three-dimensional structure of the amyloid conformer [27, 28]. The steric information can then be transferred to “daughter” molecular entities through the template-assisted conformational replication cycles generating replicas of the spatially altered amyloid conformer [11]. Fragmentation and the formation of new seeds characterize the primary replication process; the importance of fragment recycling in the prebiotic evolutive processes has been emphasized [29]. The oligomeric phase of the replication process appears to be essential for the inscription of environmental information.

**Fig. 2:**
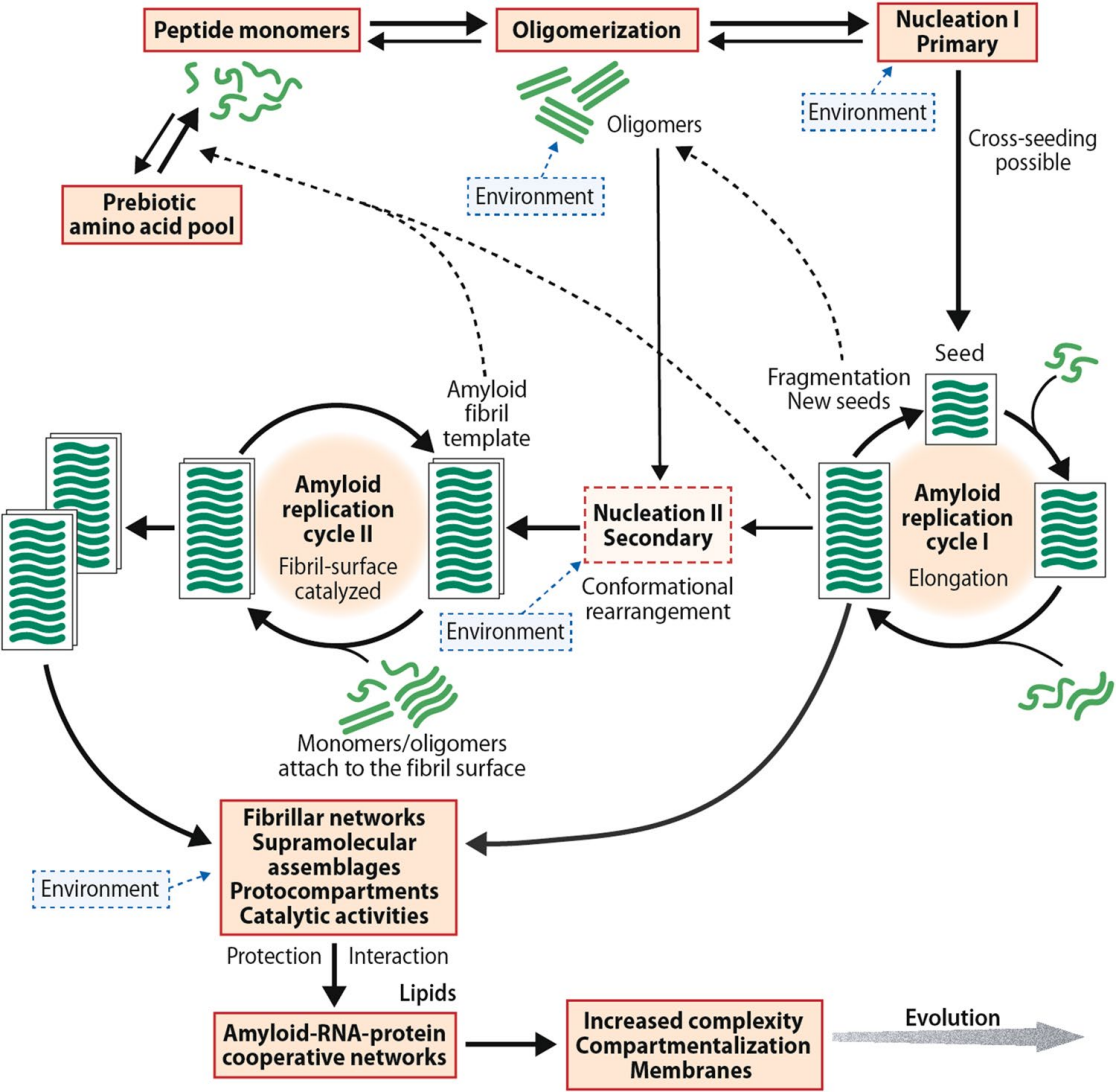
Schematic representation of the amyloid model of the origin of life. The figure outlines the proposed pathway of one type of peptide monomer from a prebiotic mixture of various protopeptides. The nucleation-dependent replication system is in-put sensitive, chiroselective, and error correcting. An initial slow nucleation process is followed by a fast polymerization phase where peptide monomers are added the growing end of the protofilament. Fragmentation generates new seeds that can initiate repeated replication cycles. The same peptide monomer can give rise to different amyloid structures and molecular rearrangements are possible. Specific conformational changes can be replicated in the fibril/protofibril-catalyzed cycle II. Amyloid is also able to direct the synthesis of its own constituent peptides. The β-sheet conformers and ribonucleotides interact dynamically and cooperatively, and the amyloid-based supramolecular fibrillar assemblies can function as a primitive metabolic apparatus catalyzing the formation metabolite precursors. The model does not exclude the possibility an extraterrestrial origin of the primordial amino acids or a contribution of extraterrestrial amino acids to the terrestrial prebiotic amino acid pool. References are given in the text

A schematic representation of the replication cycles is shown in Fig. 2. Importantly, two different nucleation mechanisms exist. A primary one, that typically involves a short seed, and a secondary, fibril surface-catalyzed step, which occurs when a small, but critical amyloid concentration has been achieved [30–33]. The fibril-catalyzed step is characterized by an initial fast docking phase followed by a slow structural rearrangement locking phase [34]. From a prebiotic perspective, the demonstrations of template-assisted ligation of fibrillogenic peptides from two shorter building blocks [23–25] and of amyloid-directed synthesis of its constituent peptides from amino acids [26] are important.

## Adaptive and enantioselective amyloids

A distinctive feature of amyloid formation is that the same peptide monomer can generate functionally and structurally different amyloid conformers of which one or several can propagate and make new copies of itself/themselves [35, 36]. This ability is important with respect to the evolvability of the system: evolution requires variation. The replicating system can adapt to even small changes in the external milieu. The environmentally induced fine-tuned changes in the amyloid architecture can then be replicated and the pool of the fittest variants can expand. In this model, the environmentally less suitable conformers are degraded and the released monomers/oligomers are reutilized in the replication cycles [11].

The amyloid polymorphs differ from each other in the architecture of the β-strands within and between the β-sheets and in the stacking of the β-plates, as well as in filament length. The number of protofilaments per fibril and the degree of twisting of the fibril can also vary. The β-sheets are tightly packed without water molecules between them; hydrogen bonds, van der Waals forces, and electrostatic polarization hold the zippers together [13, 18, 37]. Typically, amyloid is found as a homochiral structure which has been explained by a poorer spatial fit of heterochiral structures [38, 39]. The high enantioselectivity of the molecular recognition of β-sheets is likely to be relevant to the question of the origin of biological homochirality [40, 41]. In a prebiotic setting even a minute enantiomeric precursor imbalance [42, 43], if present, could be amplified in the template-directed chiroselective β-sheet self-replication cycles, explaining both the amplification and the chiral transmission steps of terrestrial homochirality.

## Catalytic amyloids

The catalytic activity of the peptide-based β-sheet assemblies is an important aspect of the amyloid world model. Amyloids not only catalyze their own formation, but they are able to catalyze other chemical reactions too. Rufo et al. [16] showed that small, 7-residue amyloid-forming peptides form efficient catalysts of ester hydrolysis. Other studies have demonstrated amyloid-related aldolase [17, 19], ATP-ase [44], and carbonic anhydrase [22] activities, as well as copper-mediated oxygen activation [45]. The catalytic functions are fibril/protofibril-dependent: the corresponding nonaggregated peptides are catalytically inactive. Another feature is the metal dependence of several amyloid catalysts; metal ions both stabilize the fibrillar structure and shape ligand geometry. In a recent study, Lee et al. [46] determined the structure of a metalloamyloid esterase catalyst by solid-state NMR. The peptide formed parallel β-sheets that assembled into stacked bilayers with alternating hydrophobic and polar interphases. The hydrophobic interphase was stabilized by apolar side chains, whereas the polar interphase contained zinc-binding histidines. In another recent study, Omosun et al. [19] examined the catalytic activities of peptides assembling into well-defined amyloid nanotubes. They found that the density and proximity of the extended arrays of chiroselective catalytic sites accomplished template-assisted polymerization of new polymers. The depth of the co-linear cross-β grooves of a heptapeptide assembling in antiparallel in-register β-strands was shown to be important for the retro-aldol activity; the shallower grooves limited substrate binding and diminished catalytic activity. Though the total efficiency of the catalytic activities of the cross-β assemblies is, in most cases, only moderate and the catalytic repertoire limited, the amyloid supramolecular network can, in a way, be regarded as a primitive metabolic apparatus with protoenzymatic activities.

The prebiotic relevance of the β-sheet networks and assemblies was recently highlighted by Tena-Solsona et al. [17] who provided evidence for emergent catalytic behavior of self-assembled low-molecular-weight peptide aggregates, and by Nanda et al. [21] who demonstrated error correction within replication networks through the emergence of short polymers exhibiting selective autocatalytic properties. Very recently, Rout et al. [26] showed that an amyloid can direct the sequence-, regio-, and stereo-selective condensation of amino acid synthesis, and that the templating reaction is stable over a wide range of pH (5.6–8.6) and temperature (25–90 °C). This is of particular interest from the perspective of early molecular evolution as it demonstrates that an amyloid formed from short peptides can direct the synthesis of its own constituent peptides under plausible prebiotic Earth conditions.

## Emergence of cooperative networks

**Fig. 3:**
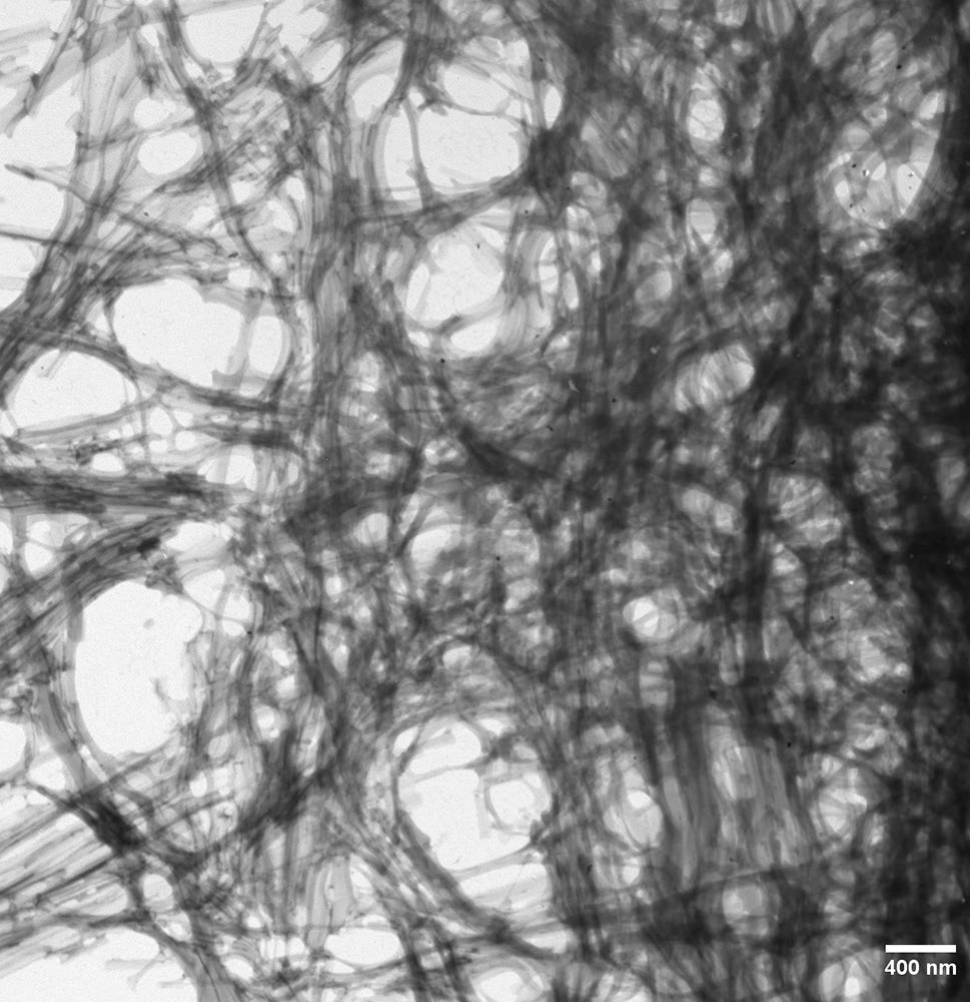
Electron micrograph of a polymorphic fibrillar amyloid network self-assembled from a prebiotically relevant 9-mer peptide (EGGSVVAAD) in aqueous environment. Experimental conditions were as described in Ref. [10]

The amyloid model fulfils key criteria of a valid origin-of-life theory, i.e., the requirements of replication, information transfer, and variation. By repeated replication cycles, the amyloid conformers can generate a variety of polymorphic fibrillar networks (Fig. 3) and structures such as nanotubes, nanospheres, and hydrogels [47]. The fibrillar assemblies can act as scaffolds for the amyloids themselves and for RNA, protein, and lipids [48–55]. They could also, under harsh prebiotic conditions, provide protection for nucleobases and natively folded peptides/proteins. Notably, extant organisms utilize amyloid structures for protection against environmental hazards [56–58] and for driving compartment formation [59].

The role of amyloid in the putative transition of the pristine amyloid world to an amyloid–RNA–protein world is not limited to scaffolding and protection; the interactions of amyloid with ribonucleotides and protein are both cooperative and dynamic. Fibrillar amyloid can act as an auto-catalyzing surface [34, 60–62] and, in addition to promoting its own formation, can, in a similar manner to that of clay and other mineral surfaces [63, 64], bind nucleic acids and enhance their polymerization, which, in turn, can promote amyloid production [15, 65–68]. Moreover, the amyloid–nucleic acid complexes may enhance nucleic acid hybridization [65]. With respect to compartmentalization and membrane formation, amyloid–lipid interactions are of key importance [50, 52, 55, 69, 70]. Amyloidogenesis is promoted by lipid interphases that lead to accelerated fibrillar network formation and the recruitment of both nucleic acids and lipids generating feed-back loops. The complex interactions between amyloids and ribonucleotides are also reflected in current biology: amyloid-forming proteins are overrepresented among the factors that modulate the transcription, translation, and storage of RNA [71], and amyloid-like aggregation has been implicated in the formation of both RNA granules [72] and P-bodies [73]. In certain instances, protein’s amyloidogenic properties and RNA-modulating activity are associated with the occurrence of glutamine/asparagine-rich sequence motifs.

The transition from an amyloid world to an amyloid–RNA–protein world is likely to have evolved over a long period of time. However, the amyloid model [8, 11, 74] is also compatible with a gradually coevolving RNA–protein world [75, 76]. The scaffolding properties, protection, and protocompartments provided by amyloid supramolecular structures in combination with the cooperative interactions and interdependence between amyloid, ribonucleotides, and protein are likely to have constituted a driving force in prebiotic molecular evolution.

## The prion connection

Prions are self-propagating infectious protein-based agents that can cause severe neurodegenerative disease. The prion hypothesis postulates that a misfolded form of the prion protein is the causative and transmissible agent of prion disorders [77]. Most, if not all, of the extraordinary characteristics of prions, such as protease-resistance, thermal stability, transmissibility, and strain specificity, are closely related to the amyloid fold [12, 14, 35, 78, 79]. The conversion of the prion protein into the infectious form involves a conformational rearrangement of the protein that generates an amyloid structure, and the propagation of the prions occurs via a template-seeded replication mechanism very similar to that of amyloids in general [12, 78, 80]. In addition to disease-related prions, a number of functional prions that exploit the amyloid fold for evolutionarily selected biological processes have been identified [81]. These processes include polyamine regulation [82], signal transduction [83, 84], regulation of gametogenesis [85], hormone storage [86], epigenetic inheritance [87], and memory persistence [88].

The amyloidogenic regions of prions are evolutionarily conserved, and in many instances represented by short, low-complexity sequences enriched in glutamine/asparagine domains [89, 90]. Importantly, a marked homology exists between the amino acid sequences of peptides preferentially produced in the salt-induced peptide formation reaction simulating early Earth conditions and the sequences of known prions [91]. Extant prions, and their amyloid-based functional entities, may represent a relic of the pristine β-sheet-based information system, and the amyloid fold may represent the first functional protein fold [8, 74, 91–93].

## Genetics before genetics

Information transfer on the early Earth for about 4000 million years ago occurred, according to the amyloid hypothesis, by means of a β-sheet peptide-based prion-like amyloid system in which environmentally derived information encrypted in the β-sheet zipper structure was transmitted by a templated conformational self-replication mechanism to “daughter” amyloid entities [8, 11, 74]. Recognition was mediated by amino acid side chain complementarity and coding by the β-sheet zipper structure [13, 18, 94]. The proposed system is characterized by both robustness and variability: the replication cycles are able to produce optimized stable molecular variants for evolutive forces to act on. From a primordial pool of random uncoded short protopeptides, the adaptive template-directed chiroselective and error correcting replication cycles generated amyloids that represented the first “coded” peptide polymers. Direct chemical interaction between amino acids/peptides and ribonucleotides in the primordial environment was probably important the evolution of the genetic code [95, 96].

The amyloid model emphasizes the importance of the cooperative interactions between amyloid and ribonucleotides and of the transition from a primitive β-sheet world to a more complex amyloid–RNA–protein world, a transition that was necessary from both an evolutionary and informational point of view: The information content of the β-sheet system, though potentially large, is very limited when compared to the virtually unlimited information content of a nucleic acid-based genetic system [13]. The β-system allows, on the other hand, for more rapid responses to environmental changes which would likely have been an advantage during early molecular evolution.

## The amyloid hypothesis versus peptide/protein-first hypotheses

Though based on amino acids and peptides, there are fundamental differences between the amyloid world hypothesis [8, 11] and the peptide-/protein-/GADV-first hypotheses of the origin of life [97–100]. The key point is conformation: the differences are based on the particular folding patterns of the molecular entities involved. The amyloid fold is structurally different from all the other protein folds and it is functionally unique. It has an extraordinarily stable molecular structure and conveys functionality even to short (e.g., 3- to 9-mer) peptides. This is in contrast to nonaggregated peptides that are likely to decompose under harsh conditions, and functionality requires longer peptide lengths. In a prebiotic setting, short functional (aggregated) peptides with a rigid structure would, obviously, have had a selective evolutionary advantage over less stable, natively folded longer peptides. Importantly, template-directed reactions in β-sheet-driven replication networks can perform error correction and lead to the enrichment of a functional polymer within prebiotically relevant mixtures [21].

## Amyloid fibril formation by prebiotically relevant peptides

It is well documented that under conditions simulating early Earth conditions, amino acids and short peptides are readily formed [101–105]. A high content of hydrophobic amino acids and the presence of alternating hydrophobic and hydrophilic residues tend to increase the β-sheet forming potential of peptide mixtures [106–111]. In a recent study, Greenwald et al. [112] specifically addressed the question of whether amyloid fibers can result from a condensation of amino acids under prebiotically plausible conditions. The study showed that fibrillar amyloid spontaneously formed under such conditions from a mixture of glycine, alanine, aspartate, and valine, all representing prebiotic “consensus” amino acids. An earlier study [10] had shown that a nonapeptide composed of six of the most abundantly produced amino acids in experiments simulating early Earth conditions [113], and also present in carbonaceous meteorites [114], generated polymorphic amyloid networks in an aqueous solution at temperatures likely to have existed on the primitive Earth. Moreover, intriguingly, a marked amino acid sequence homology has been observed between experimentally produced prebiotic peptides and extant amyloid-forming prions [91]. These findings are in line with the view that amyloids could have been formed and existed under early Earth conditions.

## Toward life

A crucial stage in the origin of life was the emergence of the first informational molecular entity that was able to self-organize and evolve on the primordial Earth. The amyloid model, a hybrid replication-metabolism model of the origin of life, posits that, in the pre-RNA era, information storage and transfer was based on peptide-based catalytic amyloids. The template-assisted conformational replication cycles generate a variety of amyloid polymorphs on which evolutive forces can act. Amyloid, RNA, and protein interact dynamically and cooperatively, and the amyloid assemblages can function as primitive metabolic entities catalyzing the formation of simple metabolite precursors.

In conclusion, the emergence of an amyloid-based pristine in-put sensitive, chiroselective, and error correcting information-processing system, and the evolvement of mutualistic networks were, arguably, of essential importance in the dynamic processes that led to increased complexity, organization, compartmentalization, and, eventually, the origin of life.
